# Supplementary material for: Genome-wide excision repair in Arabidopsis is coupled to transcription and reflects circadian gene expression patterns
Source: Nat Commun. 2018 Apr 17;9:1503. doi: 10.1038/s41467-018-03922-5 (PMC5904149; doi:10.1038/s41467-018-03922-5)
Supplement: Supplementary file 1 — Supplementary Information [file 41467_2018_3922_MOESM1_ESM.pdf]

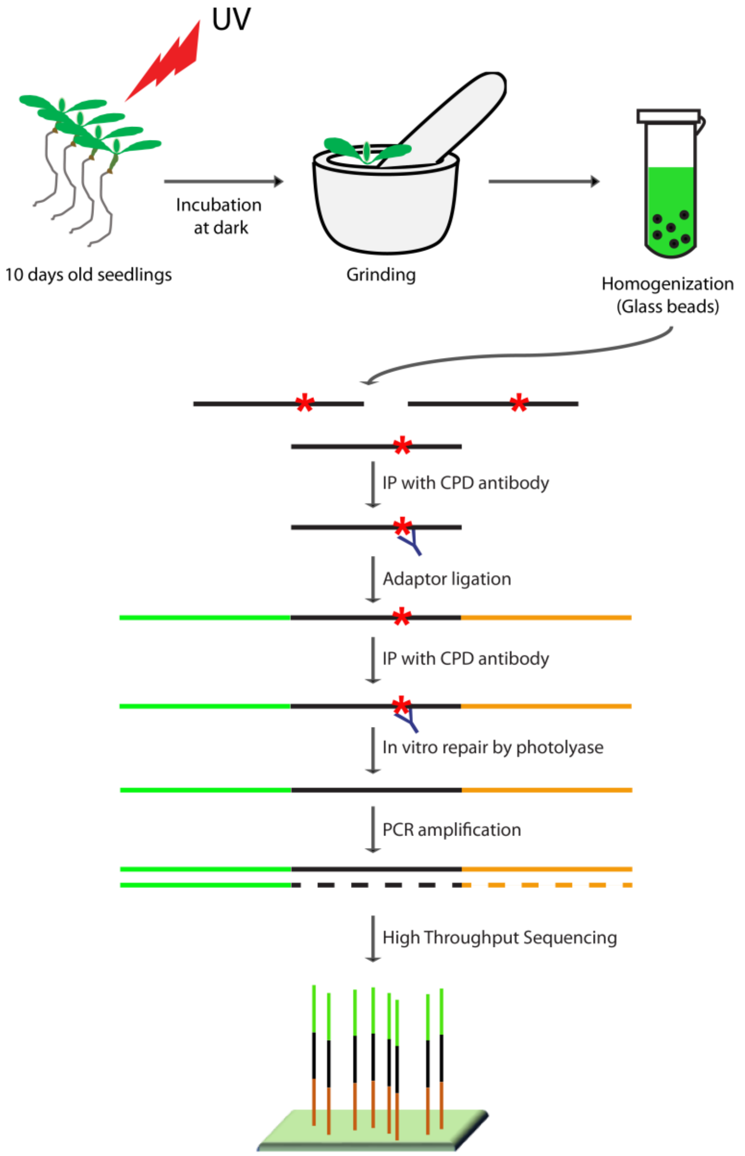

Supplementary Figure 1 | XR-seq procedure. 10-day-old seedlings were exposed to 120 J/m2 UV (254-nm), and harvested 30 min after UV exposure. The seedlings were ground using mortar and pestle, and homogenized. Excision products were isolated by immunoprecipitation (IP) with anti-CPD-DNA antibody. After ligation of 5'and 3' adapters, a second IP with anti-CPD-DNA antibody was performed. UV lesions were reversed by photolyase treatment. The fragments were amplified by PCR, and analyzed by Next Generation Sequencing.

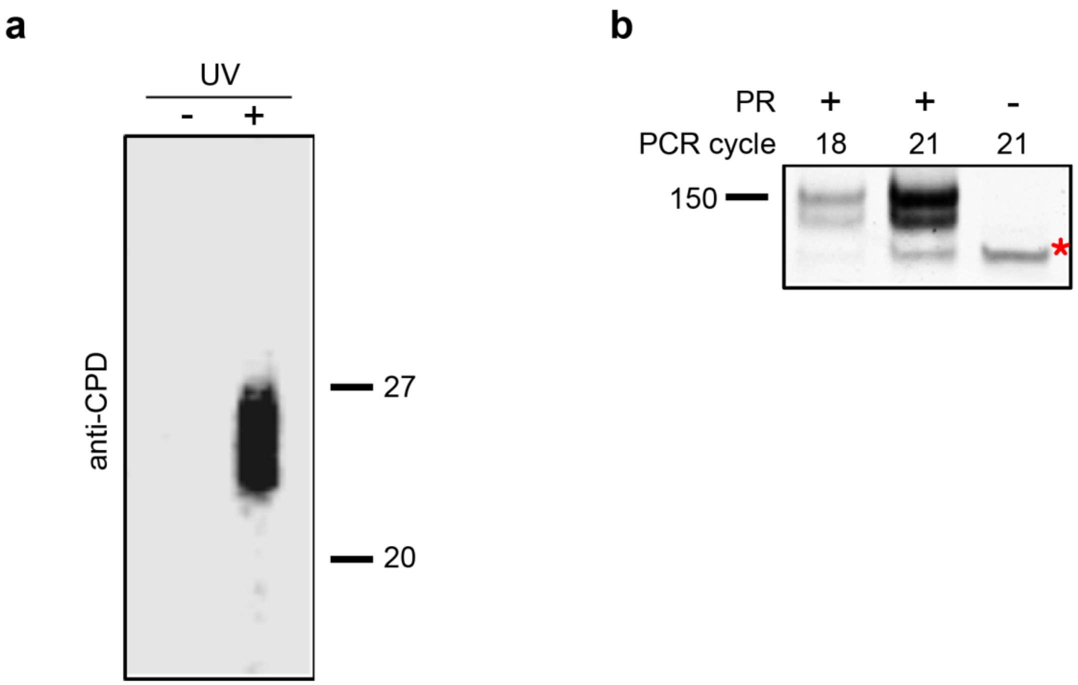

Supplementary Figure 2 | Nucleotide excision repair products in *Arabidopsis*. a, Detection of CPD-containing excision products in UV-irradiated seedlings by immunoprecipitation with anti-CPD-DNA antibody. **b,** Polyacrylamide gel showing the XR-seq dsDNA library of the excision repair products after adapter ligation, photoreactivation (PR), and PCR. The asterisk indicates a non-specific band.

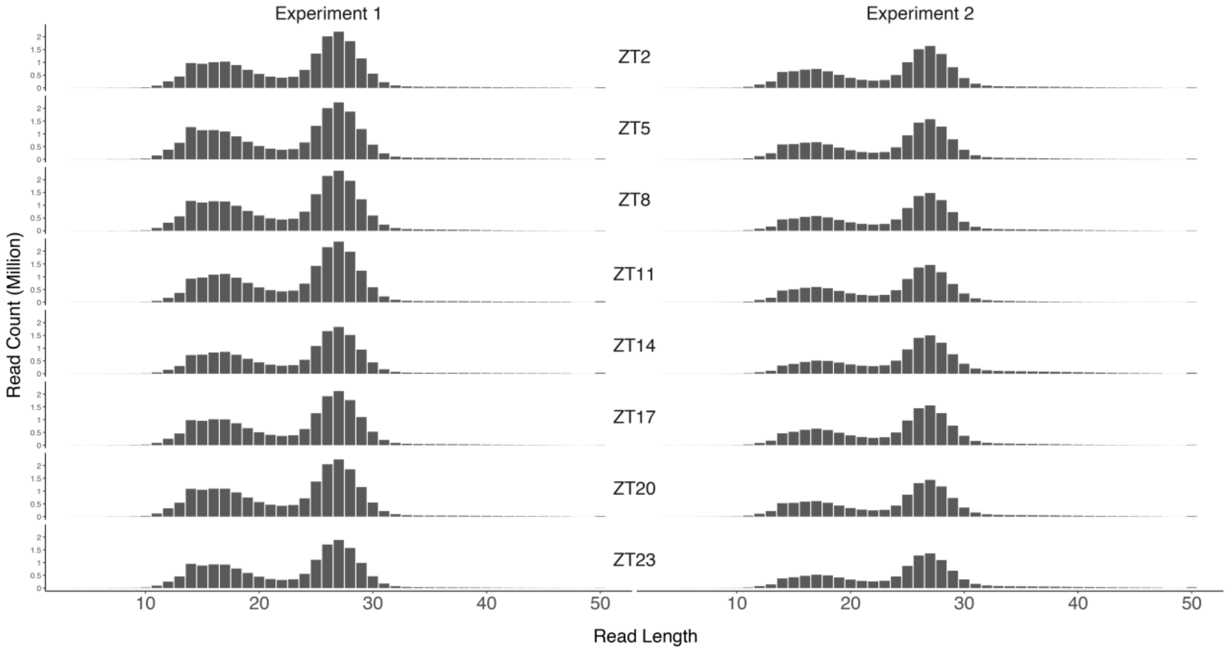

Supplementary Figure 3 | Read length distribution of each XR-seq sample. Distributions of the XR-seq reads are plotted after adapter removal. Two populations of the read lengths are observed. The long read (~27 nt) population is the expected intact excised oligomers whereas the short-read population is degraded. The first and second experiments were sequenced using Illumina HiSeq4000 and HiSeq2500, respectively. The sequencing platform difference is the reason for fewer reads for the second set. ZT2-ZT23 stand for different circadian time points.

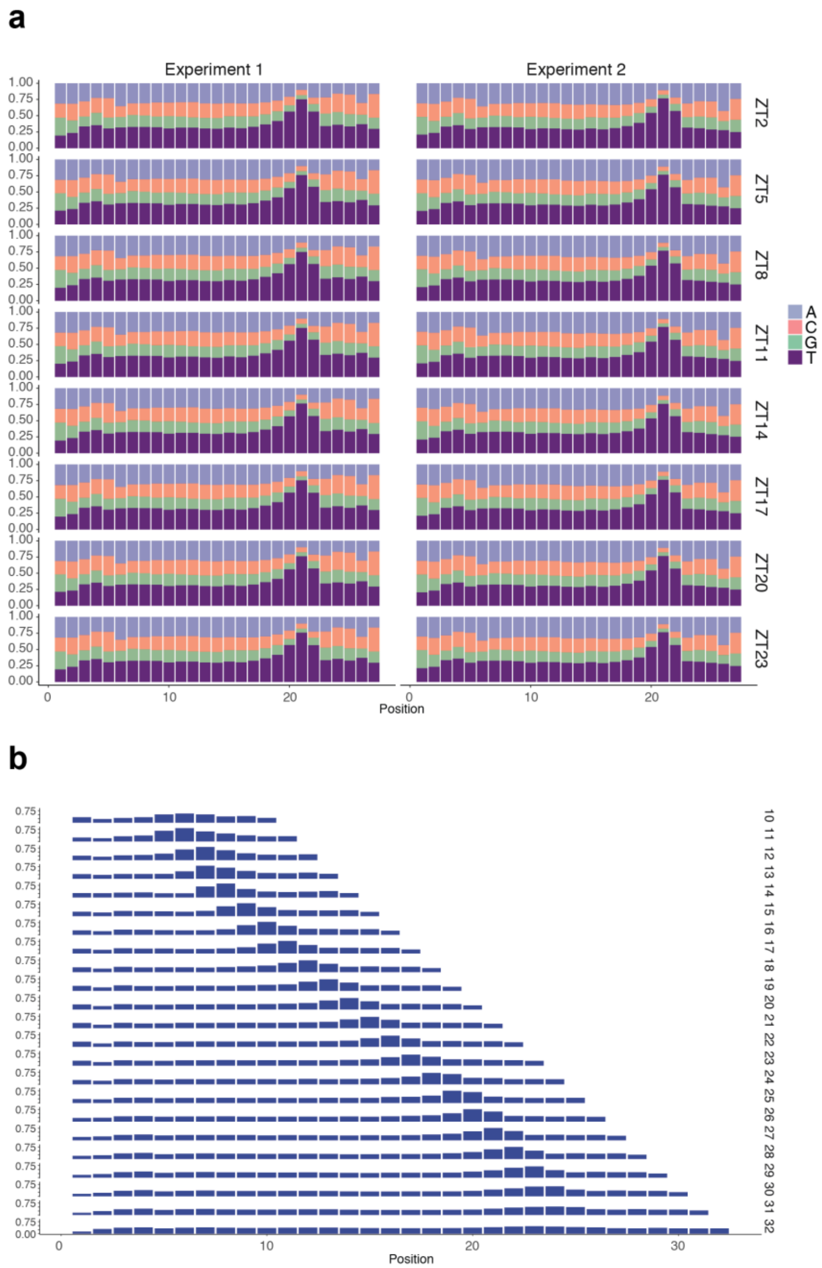

Supplementary Figure 4 | Nucleotide content of the excised oligomers. a, Nucleotide frequencies of the excised products are plotted for the 27 nt oligomers from each XR-seq sample. ZT2-ZT23 stand for different circadian time points. **b,** Thymine frequencies for combined samples are shown for the read lengths from 10 to 32. The thymine enrichment is observed 6-8 nt from 3’ end for all oligomers between from 11 to 30 nt lengths, which suggests that the excised oligomers are degraded by 5’-to-3’ exonuclease activity.

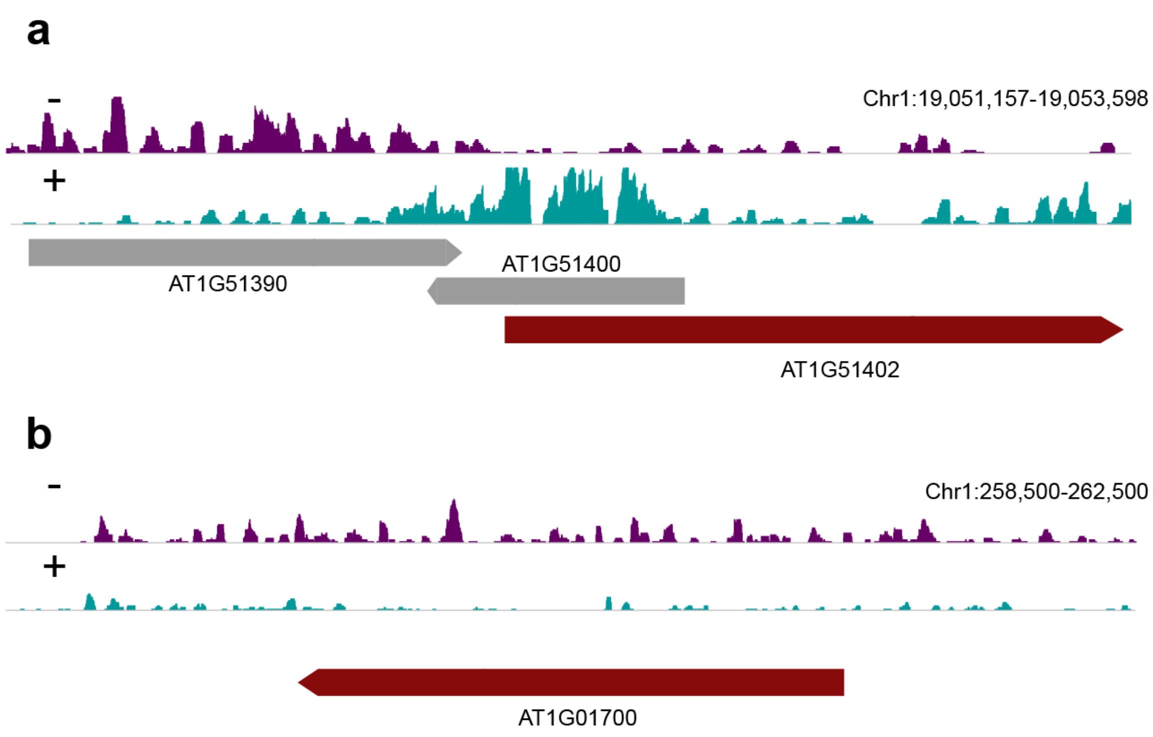

Supplementary Figure 5 | Repair preference on the non-transcribed strand. Potential reasons why a small percentage of *Arabidopsis* genes have higher NTS repair relative to TS are illustrated: **a,** overlapping genes and **b,** anti-sense transcription. The illustrated anti-sense transcription is currently not annotated.

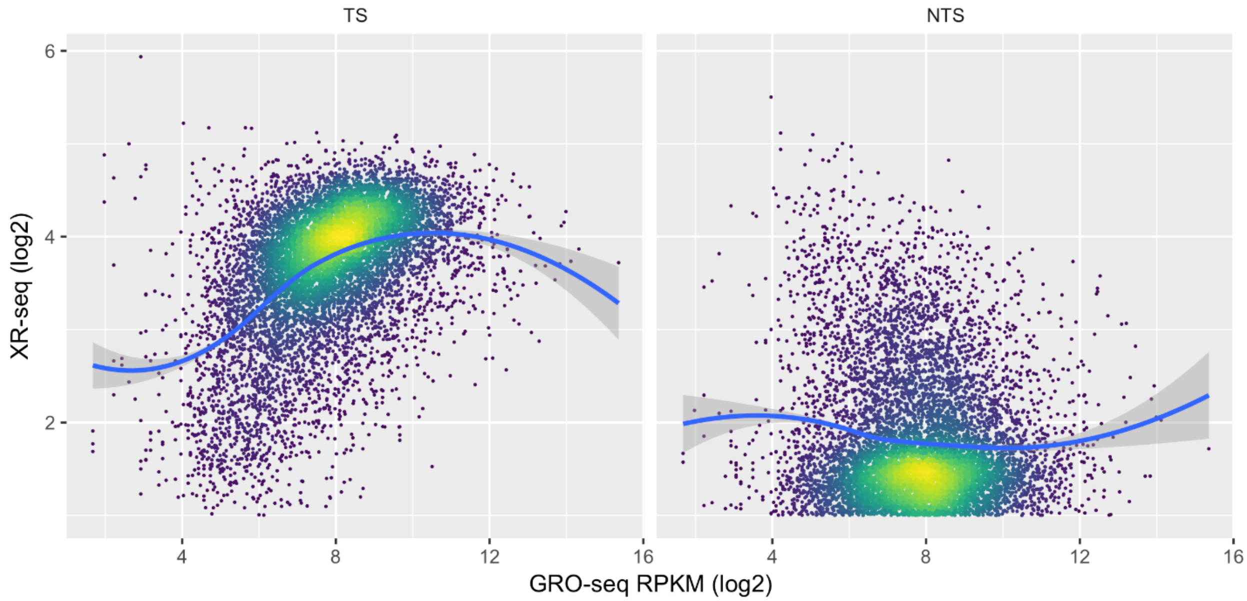

Supplementary Figure 6 | Correlation between transcription and TCR. Each transcribed gene is shown as a point in the function of XR-seq (y-axis, for two strands; TS and NTS) and GRO-seq (x-axis). Values represent the log2(RPKM + 1) values. The genes with no mapped read (XR-seq or GRO-seq) were removed. The LOESS curve was fitted (blue). Color of each dot represents the density from low (purple) to high (yellow).

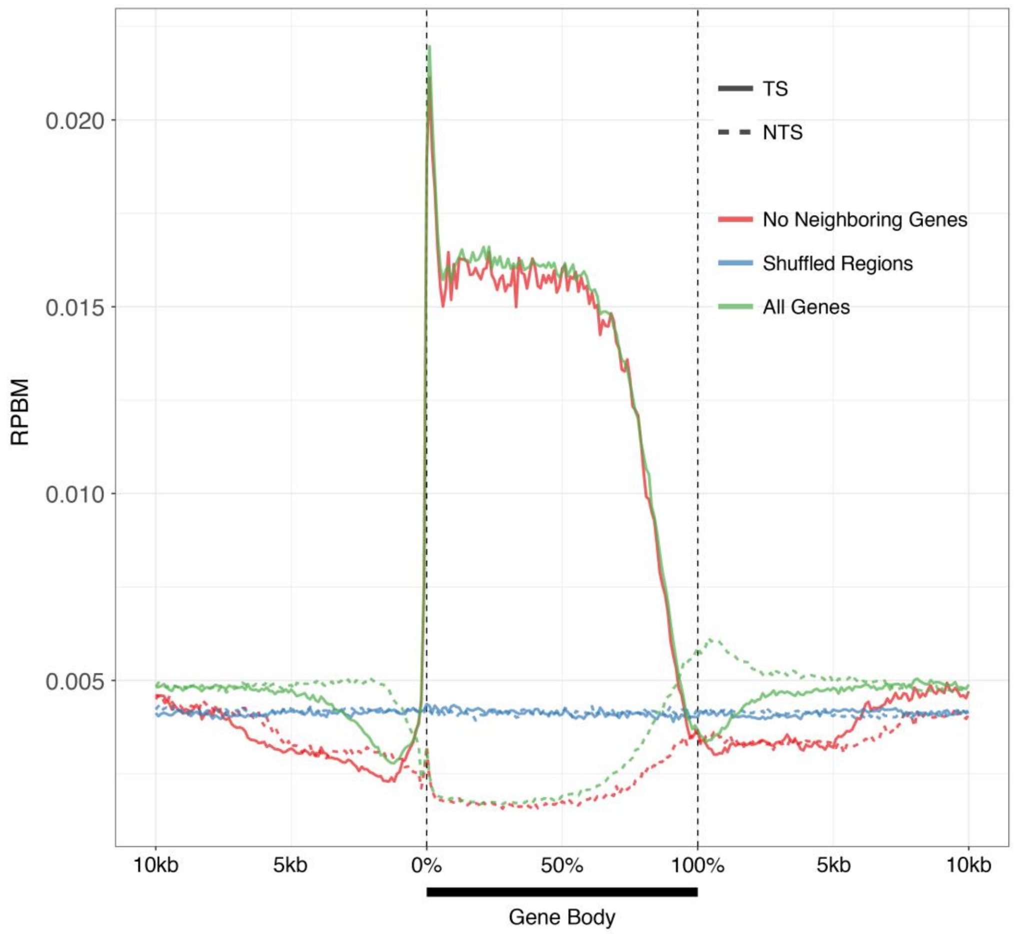

Supplementary Figure 7 | TCR profiles of genes with no neighboring annotated transcription within 5kb proximity. The level of repair (≈0.004 RPBM) among all shuffled regions (blue) provides a baseline that includes global repair and TCR. The level of repair in the NTS of gene bodies (≈0.0035 RPBM red, green dotted lines) is considered a truer measure of baseline global repair in the absence of TCR. This is because while among all genes, NTS repair returns to the ≈0.004 RPBM level immediately upstream and downstream of the gene body, among genes with no known nearby gene neighbors, NTS repair remains near the gene body-level immediately upstream and downstream. The signal elevation from 5 to 10kb is another indicator of the neighbor transcription effect on flanking repair. Thus, intergenic regions with no TCR are relatively uncommon in the compact *Arabidopsis* genome.

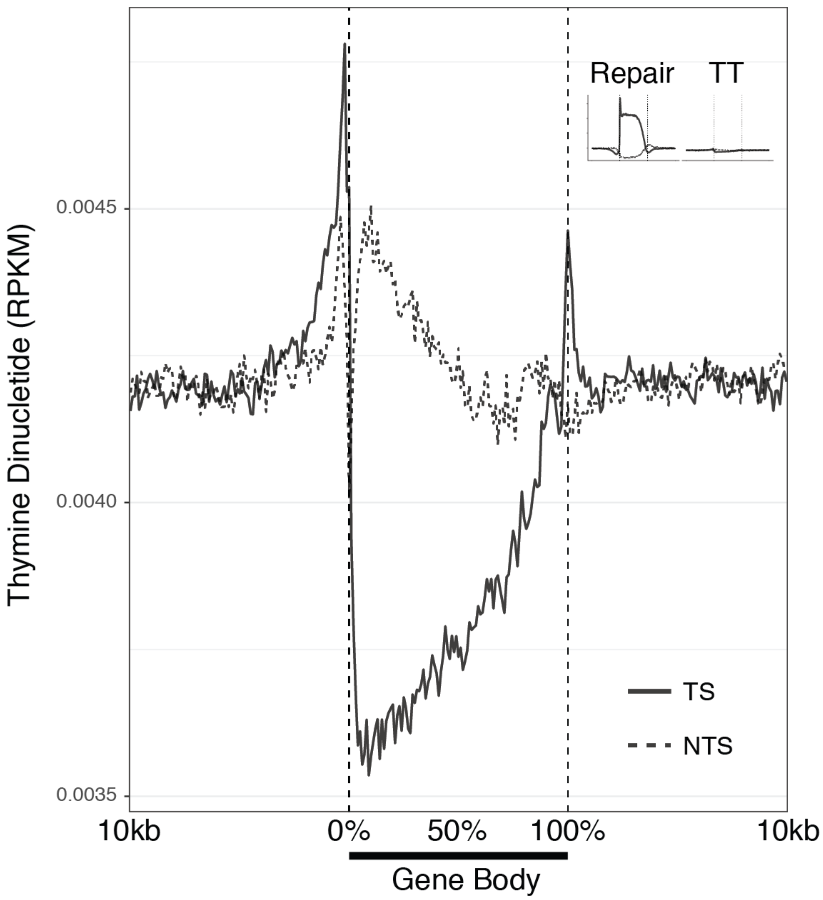

Supplementary Figure 8 | Dithymine (TT) content of the transcribed and non-transcribed strands. In order to evaluate the TS repair preference over NTS, sequence context bias was measured. In fact, the TS dithymine levels are lower than the NTS. The inset shows the minor effect of sequence context relative to the TS repair preference on the same scale.

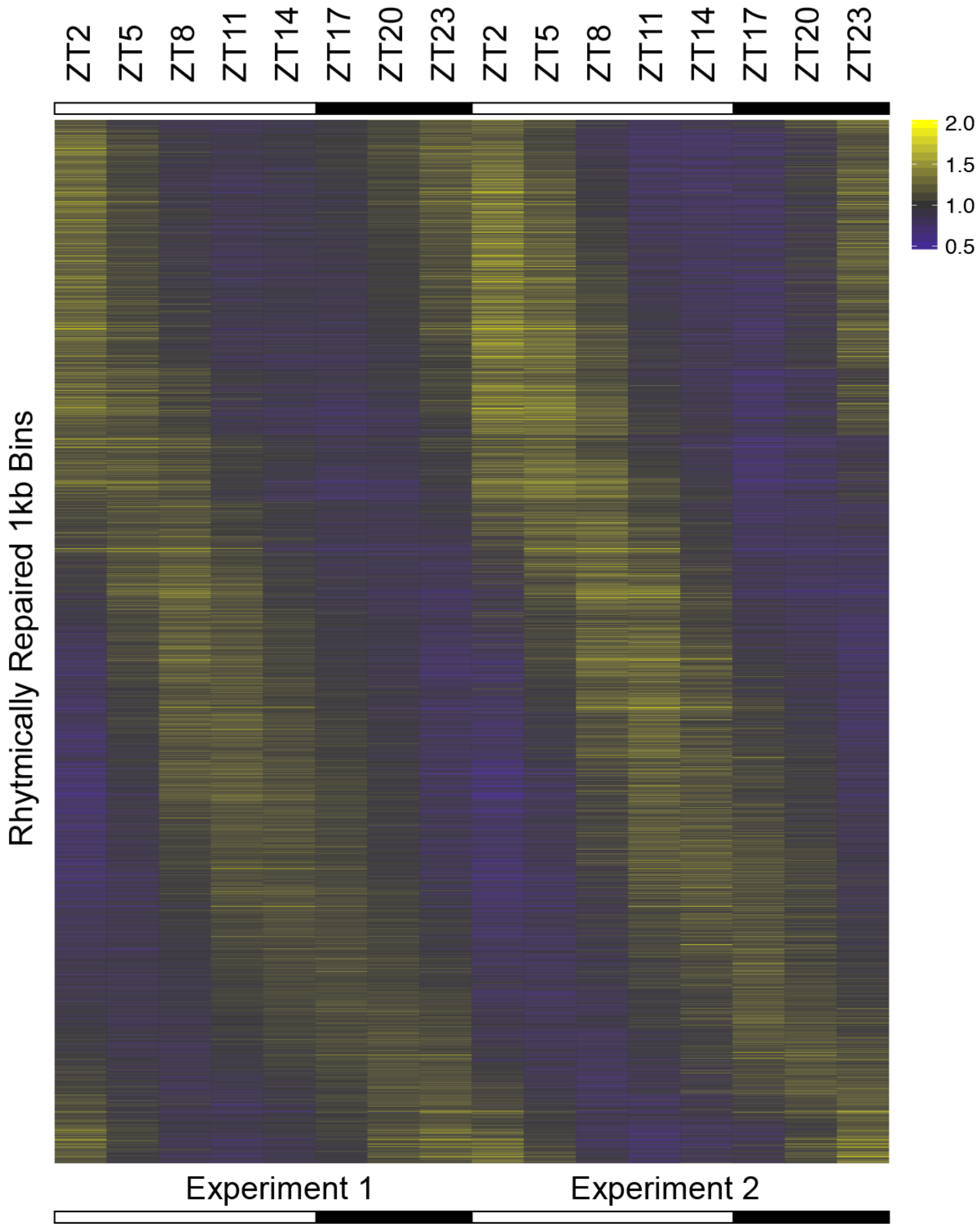

Supplementary Figure 9 | Repair oscillation in the entire genome. The entire genome was divided into 1kb bins, and repair levels (both strands) were computed for each bin. Metacycle software was used to reveal the bins with repair oscillation. Rhythmic bins are plotted as a heatmap. The color reflects the ratio of the observed to median repair value for each bin. With the thresholds used (see methods) to decide whether a bin has an oscillation, ~6 Mb of the *Arabidopsis* genome exhibited rhythmic excision repair, which corresponds to ~5% of the genome.

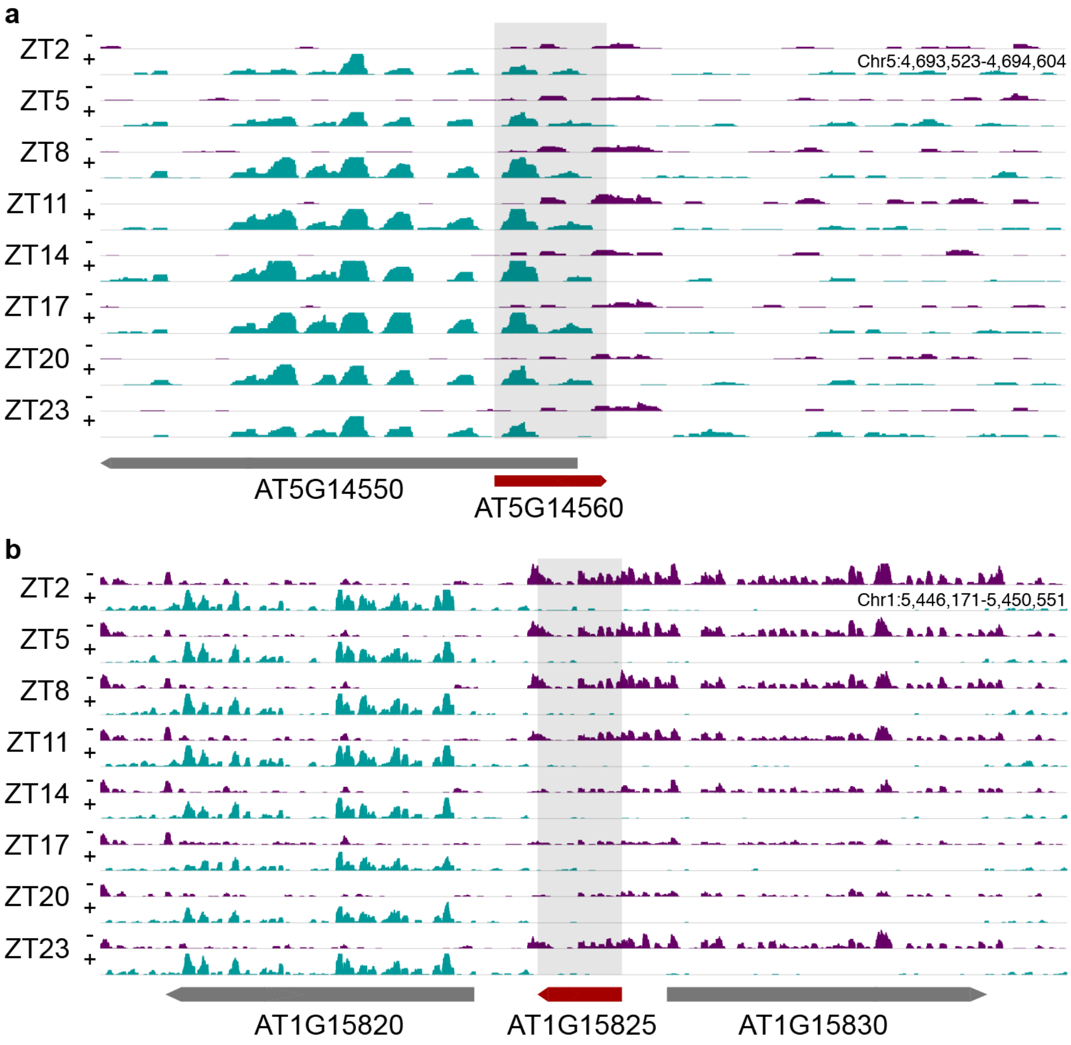

Supplementary Figure 10 | Repair oscillation on the non-transcribed strand. A few genes exhibited rhythmic NTS repair which may be due to TRC of an overlapping gene with an opposite orientation, as in **a,**, or due to anti-sense transcription and TRC within the gene body, as in **b**.

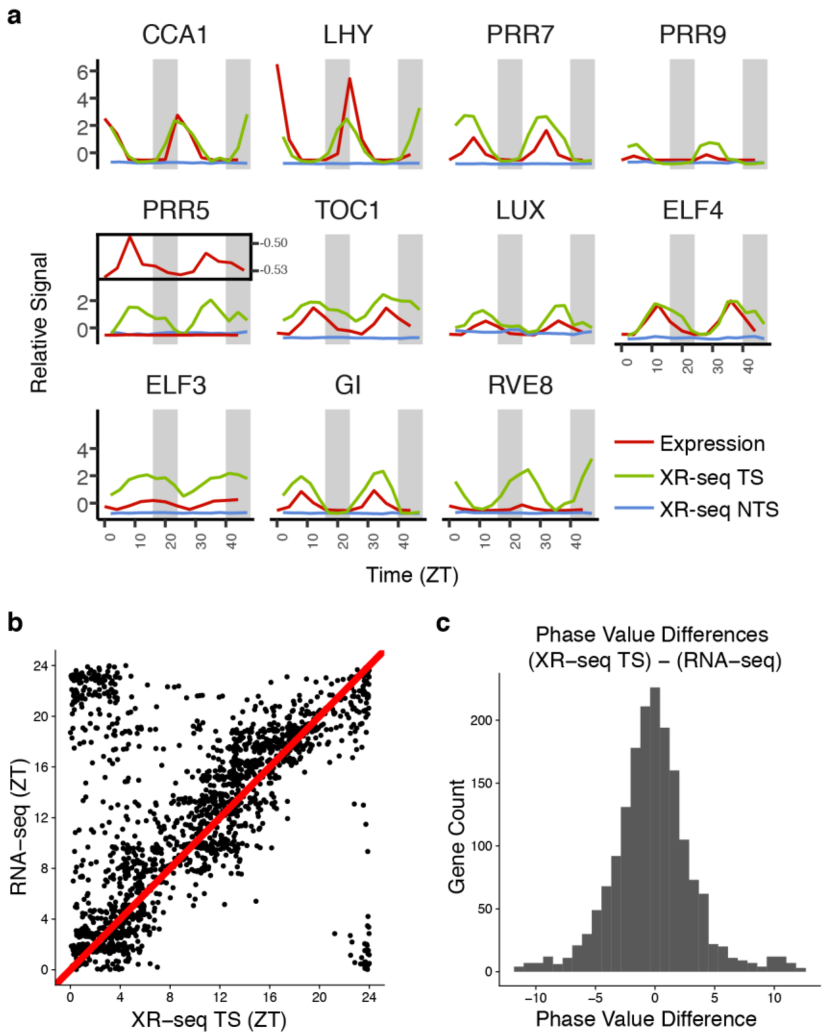

Supplementary Figure 11 | Correlation of rhythms of gene expression and rhythms of TS repair. a, The expression rhythms of the core clock genes are in agreement with the TCR rhythms. The repair and expression values were scaled independently to be visualized at a comparable scale (y-axis). **b,** Peak expression (RNA-seq, y-axis) values and peak TCR (TS XR-seq, x-axis) values of the 1754 significantly rhythmic genes are correlated. The red line corresponds to the identity line (x=y). The genes grouped at top-left and bottom-right are due to the circularity of the 24-hour rhythms. **c,** The distribution of phase shifts between peak expression and peak transcription-coupled repair is shown as a histogram. The phase difference values were processed to remove the circularity effect, for example ZT23 – ZT1 is represented as -2.

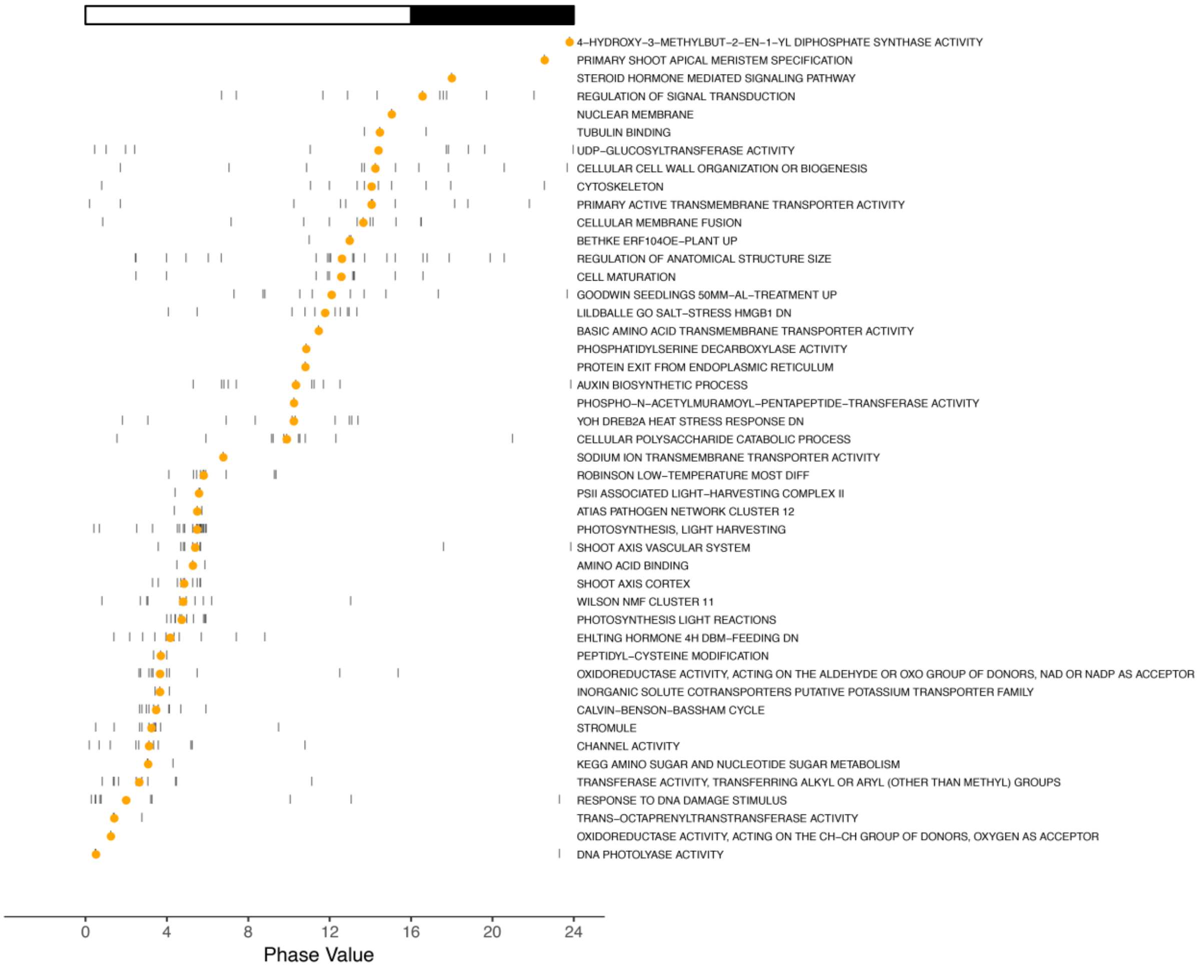

Supplementary Figure 12 | A comprehensive phase set enrichment analysis. Phase set enrichment analysis was performed to identify pathways of gene groups that have clustered phase values. The Kuiper test was used to decide whether cumulative distributions between the gene set of a specific category and all oscillating genes (n=1453) are different.
